# Urinary tract infections and intermittent catheterization among patients with spinal cord injury in Chinese community

**DOI:** 10.1038/s41598-023-44697-0

**Published:** 2023-10-17

**Authors:** Jiawei Liu, Can Luo, Weichu Xiao, Tao Xu

**Affiliations:** Department of Rehabilitation, Tongji Hospital affiliated to Tongji Medical College of Huazhong University of Science & Technology, Wuhan, 430030 Hubei China

**Keywords:** Infectious diseases, Trauma, Spine regulation and structure, Neurological disorders, Bladder, Urogenital diseases

## Abstract

We conducted a cross-sectional study using an online questionnaire to investigate the current status of urinary tract infections (UTIs) and the use of clean intermittent catheterization (CIC) in Chinese community-based SCI patients and to explore the risk factors for UTIs in patients using CIC. Our findings suggest that the prevalence of UTIS is higher in Chinese community-based SCI patients compared with patients in medically developed countries. In addition, we found that CIC had the lower incidence of UTIs than urinary indwelling catheter (UIC) and suprapubic catheter (SPC), and that SCI patients with CIC had low rates of use and poor compliance. Further analysis indicated that most of the risk factors for UTIs in CIC patients were associated with irregular use of CIC. Therefore, we call for not only the need to provide stronger caregiver support and financial assistance to improve CIC adherence in Chinese community SCI patients, but also the establishment of a database of Chinese SCI patients in order to enhance the management of bladder emptying methods and further standardize the CIC operation in such patients, thus reducing the risk of UTIs in Chinese community SCI patients.

## Introduction

Urinary tract infections (UTIs) have been the most common urological complication in spinal cord injury (SCI) patients, prolonging hospital stays, impeding the progress of recovery, and increasing the financial burden^1–3^. A comprehensive epidemiological study conducted in the United States showed that 36% of SCI patients suffered from UTIs^4^. In a 5-year post discharge follow-up study conducted in the Netherlands, the incidence of UTIs was even higher, at 56.5–58.9%^5^. Poorly controlled UTIs could have various serious consequences, the most common of which are sepsis, acute kidney damage, and renal failure^6,7^. However, unfortunately, there exists no information database on SCI management in mainland China^8^, so it is almost impossible to obtain UTIs information on SCI patients through official database channels. Previous studies in China have mainly reported on the UTIs of in-hospital SCI patients and have focused on only a few developed provinces and cities^9–12^. Therefore, existing studies lack national or community-based reports on UTIs in Chinese SCI patients. This has led to a lack of understanding of the current status of UTIs in Chinese community-based SCI patients and consequently insufficient attention to finding ways to reduce this highly prevalent urological complication.

In addition, bladder management in patients with SCI is important as it is considered to have a significant relationship with the patient's prognosis and quality of life^13^. Since existing studies have exquisitely found that SCI impairs voluntary control of voiding and the normal reflex pathways that coordinate bladder and sphincter function^14,15^, proper bladder management is essential for the prevention of urological complications in patients with SCI^13^. Furthermore, several articles have reported that the method of bladder emptying may be a major risk factor for the development of urolithiasis in patients with SCI^16,17^. Consequently, with the help of the National Alliance for Spinal Cord Injury Patient Care, we initiated a questionnaire survey on urolithiasis-related issues covering 30 provinces in China, in the hope of understanding the current status of UTIs and bladder management in spinal cord injury patients in the Chinese community, and to find out measures to improve bladder management and reduce UTIs.

## Materials and methods

### Ethical considerations

Our study has been performed in accordance with the Declaration of Helsinki and approved by the Ethics Committee of Tongji Hospital, Tongji Medical College, Huazhong University of Science and Technology (Ethics No. TJ-IRB20210314). Subjects were informed of the purpose of this study and were fully aware of the consent prior to the investigation. The identity of the patients participating in the study was kept strictly confidential.

### Patient population

The patient data analysed in this study was obtain from a project investigating disease burden and the quality of life of Chinese patients with SCI in China. The project was initiated by the National Coalition for the Care of People with SCI and co-organized by the China Association of Persons with Physical Disability and the team from the Department of Rehabilitation of Tongji Hospital.

Inclusion criteria for the study included (1) patients diagnosed with SCI, according to the International Classification of Diseases (version 10). (2) Patients discharged from hospitals after treatment for SCI. Patient information was collected through an online questionnaire. A total of 3120 community patients with spinal cord injury in 30 provinces in China were invited to complete the questionnaire between August 3 and August 31, 2020.

We excluded (1) invalid questionnaires and questionnaires with abnormal values; and (2) questionnaires that did not explicitly state the bladder emptying method used in the last year. Our final sample consisted of 294 patients with spinal cord injury (2051 male patients and 873 female patients). The median age of the patients was 45 (17) years and the average duration of disease was 142 (186) months, as shown in Table 1.

**Table 1:** Basic characteristics of the study population.

Characteristics	Categories	Total (*n* = 2924)
		*N* (%)
Gender	Male	2051 (70.1)
	Female	873 (29.9)
Educational background	High School above	226 (7.7)
	High School and below	2698 (92.3)
Etiology	Traumatic	2463 (84.2)
	Nontraumatic	461 (15.8)
Level of nerve damage	Cervical	550 (18.8)
	Thoracic	1303 (44.6)
	Lumbar	889 (30.4)
	Sacral	182 (6.2)
Water intake (mL/24 h)	< 1000	1309 (44.8)
	1000–2000	1232 (42.1)
	> 2000	383 (13.1)
Urine output (mL/24 h)	< 1000	1311 (44.8)
	1000–2000	865 (29.6)
	> 2000	312 (10.7)
	Unclear	425 (15.0)
Upper limb function	Damaged	1140 (39.0)
	Normal	1784 (61.0)
Walking ability	Using wheelchair	2624 (89.7)
	Assisted walking	214 (7.3)
	Independent walking	86 (2.9)
Bladder emptying methods	Spontaneous voiding	557 (19.0)
	Assisted voiding^a^	637 (21.8)
	Urinary collector^b^	713 (24.4)
	CIC	623 (21.3)
	UIC	273 (9.3)
	SPC	121 (4.1)
Age (in years)^c^		45 (17)
Duration of disease (in months)^c^		142 (186)

UTIs, urinary tract infections; CIC, clean intermittent catheterization; SPC, suprapubic catheter; UIC, urinary indwelling catheter.

^a^Include Credé voiding, Valsalva voiding, and trigger reflex voiding.

^b^Include urine condoms and urinary pads/pants.

^c^For *Age* and *Duration of disease*, report the medians and interquartile range.

### Composition of the questionnaire

The questionnaire was designed with reference to international used scales such as the Neurogenic Bladder Symptom Score (NBSS)^18^, International Lower Urinary Tract Function Basic SCI Data Set^19^, and the 36-Item Short Form Health Survey (SF-36)^20^. In addition, templates of international and national research questions for patients with SCI were included.. National and international experts revised the questionnaire. The final version of the questionnaire included a total of 92 questions in the following five sections: (1) basic information, (2) bladder management, (3) UTIs, (4) complications, disease burden, and (5) quality of life.

### Definition of urinary tract infections

Question 2 in section (3) of the questionnaire, “During the past year, how many urinary tract infections (e.g., pain, fever, change in urine color and odor) have you had?”, was used to define UTIs with reference to the NBSS^18^. Patients who answered “greater than or equal to 1 time” to this question were considered to have had a UTIs, and therefore, these patients were included in the UTIs group, while those who answered “equal to 0 times” were included in the non-UTIs group. Recurrent urinary tract infections (RUTIs) are defined as having at least 3 UTIs in a year.

### Bladder emptying methods

Question 4 in section (2) of the questionnaire, “Which type of urination method have you used most frequently in the past year?”^19^ was used to define bladder emptying methods. We have defined the following six methods of bladder emptying as follows: (1) clean intermittent catheterization: CIC; (2) urinary indwelling catheter: UIC; (3) suprapubic catheter: SPC; (4) spontaneous voiding: without external forces such as hand or abdominal pressure; (5) assisted voiding: include Credé voiding, Valsalva voiding, and trigger reflex voiding; (6) urinary collector: include urine condoms and urinary pads/pants).

### Statistical methods

The questionnaire data were exported from the Questionnaire Star web page as Excel spreadsheets and then imported into IBM SPSS Statistic22 for data analysis. In univariate analysis, differences between groups were assessed using chi-square or Fisher exact tests for categorical variables. Since all continuous variables were non-normal distributed, the non-parametric Mann–Whitney U test was utilized. Logistic regression analysis was used for multivariate analysis. P < 0.05 indicates that the differences were statistically significant.

### Ethics approval and consent to participate

This study was reviewed and approved by the Ethics Committee of Tongji Hospital, Tongji Medical College, Huazhong University of Science and Technology (Ethics No. TJ-IRB20210314). The identity of the participants was kept strictly confidential, and they were fully appraised of the nature of the study and all provided informed consent before taking the survey.

## Results

**Figure 1:**
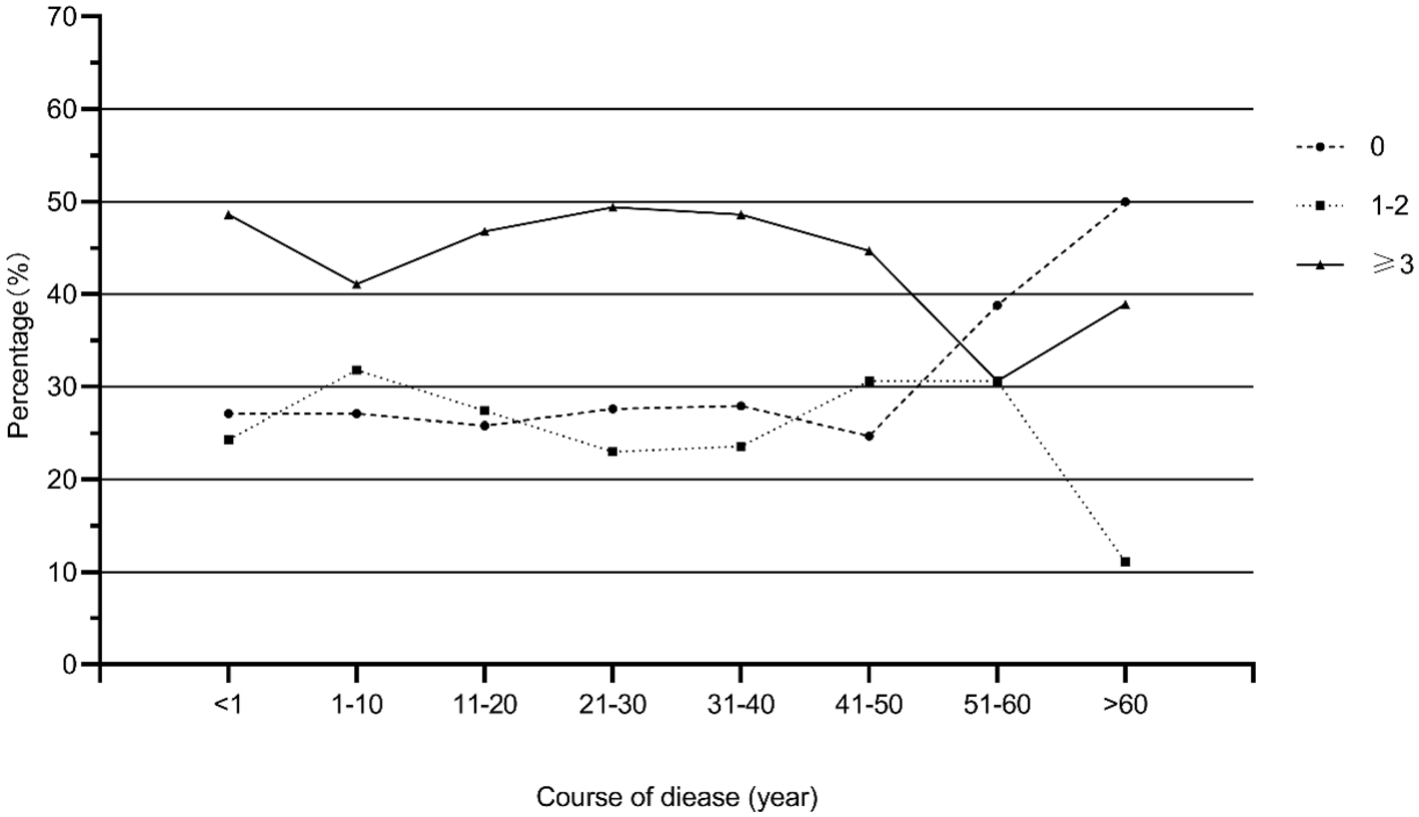
Incidence of UTls in patients with SCI in different course of disease.

**Figure 2:**
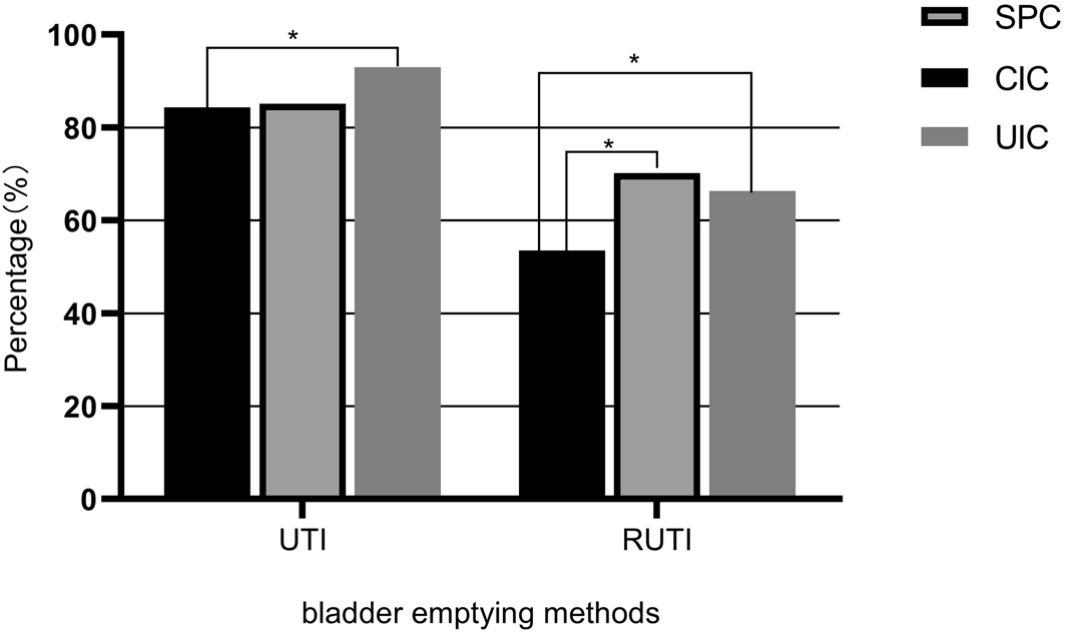
The incidence of UTI and RUTI between the groups of CIC, SPC, and UIC. UTIs, urinary tract infections; RUTIs, recurrent urinary tract infections; CIC, clean intermittent catheterization; SPC, suprapubic catheter; UIC, urinary indwelling catheter. *Statistically significant differences at P < 0.05.

Between August 2019 and August 2020, the incidence rate of UTIs and RUTIs in Chinese community SCI patients was 72.9% and 44.7%, respectively. As shown in Fig. 1, this proportion is relatively stable, maintaining a level of about 70% and 45% among patients with different duration of the disease (within 50 years of disease duration), with little fluctuation. Basic characteristics of the study population are shown in Table 1. In our sample, 19.0% of the patients used spontaneous voiding; 21.8% using used assisted voiding; 24.4% used urinary collector; 21.3% used CIC; the other two methods, UIC and SPC, are used less frequently, with UIC at 9.3% and SPC at 4.1%. Incidence and disease burden of UTIs by different bladder emptying methods are demonstrated in Table 2. There was a significant difference in the prevalence of UTIs and RUTIs among the six bladder emptying methods (P < 0.05). Patients with spontaneous voiding had the lowest incidence of UTIs and RUTIs, 44.5% and 18.5%, respectively. While the bladder emptying methods with the highest incidence of UTIs and RUTIs differed. The highest incidence of UTIs was 93% in UIC and the highest incidence of RUTIs was 70.2% in SPC. In a comparison of the incidence of UTIs and RUTIs between the groups of CIC, SPC, and UIC (Fig. 2), although there was no significant difference in the incidence of UTIs between SPC and CIC patients, the incidence of RUTIs was significantly higher in SPC patients than in CIC patients (P < 0.05), and was similar to the incidence of RUTIs in UIC patients. In terms of disease burden, there was no significant difference in the number of days of consultation and hospitalization due to uremia in one year among CIC, SPC and UIC patients (P > 0.05).

**Table 2:** Incidence and disease burden of UTIs by different bladder emptying methods.

Bladder emptying methods	Non-Catheterized(N = 1907)			Catheterized(N = 1017)			*P*
	Spontaneous voiding	Assisted voiding^a^	Urinary collector^b^	CIC	SPC	UIC	
UTIs *N* (%)	248 (44.5)	455 (71.4)	546 (76.6)	525 (84.3)	103 (85.1)	254 (93)	< 0.001
RUTIs *N* (%)	103 (18.5)	264 (41.4)	341 (47.8)	333 (53.5)	85 (70.2)	181 (66.3)	< 0.001
Number of outpatient visits for UTIs	0 (2)	2 (4)	2 (5)	3 (7)	4 (8)	4 (7)	< 0.001
Number of hospitalizations for UTIs	0 (8)	2 (20)	2 (20)	12 (55)	13 (31)	15 (60)	< 0.001

UTIs, urinary tract infections; RUTIs, recurrent urinary tract infections; CIC, clean intermittent catheterization; SPC, suprapubic catheter; UIC, urinary indwelling catheter.

^a^Include Credé voiding, Valsalva voiding, and trigger reflex voiding.

^b^Include urine condoms and urinary pads/pants.

The method of bladder emptying used by patients with different disease duration also varied (P < 0.001). Table 3 shows that the percentage of patients using CIC was 51.4% in the group with a disease duration of < 1 year and decreased to 29.6% in patients with a disease duration of 1–10 years. The percentage of CIC use further decreased to 14.3% in patients with a disease duration of > 10 years, which was significantly different from patients with a disease duration of < 1 year (P < 0.05). Unusually, the percentage of SPC and UIC use did not change significantly with disease duration, and there was no significant difference among the three groups with different disease durations. In contrast, the percentage of spontaneous voiding, assisted voiding, and urinary collectors was proportionally higher in patients with a disease duration of > 10 years compared with those with a disease duration of < 1 year (P < 0.05).

**Table 3:** The relationship between bladder emptying methods and duration of the disease.

Bladder emptying methods	< 1 year	1–10 years	> 10 years	*P*
	*N* (%)	*N* (%)	*N* (%)	
Spontaneous voiding	5 (7.1)	174 (14.8)	378 (22.5)	< 0.001
Assisted voiding^a^	8 (11.4)	222 (18.9)	407 (24.2)	
Urinary collector^b^	7 (10.0)	284 (24.2)	422 (25.1)	
CIC	36 (51.4)	347 (29.6)	240 (14.3)	
UIC	12 (17.1)	107 (9.1)	154 (9.2)	
SPC	2 (2.9)	39 (3.3)	80 (4.8)	

UTIs, urinary tract infections; CIC, clean intermittent catheterization; SPC, suprapubic catheter; UIC, urinary indwelling catheter.

^a^Include Credé voiding, Valsalva voiding, and trigger reflex voiding.

^b^Include urine condoms and urinary pads/pants.

**Figure 3:**
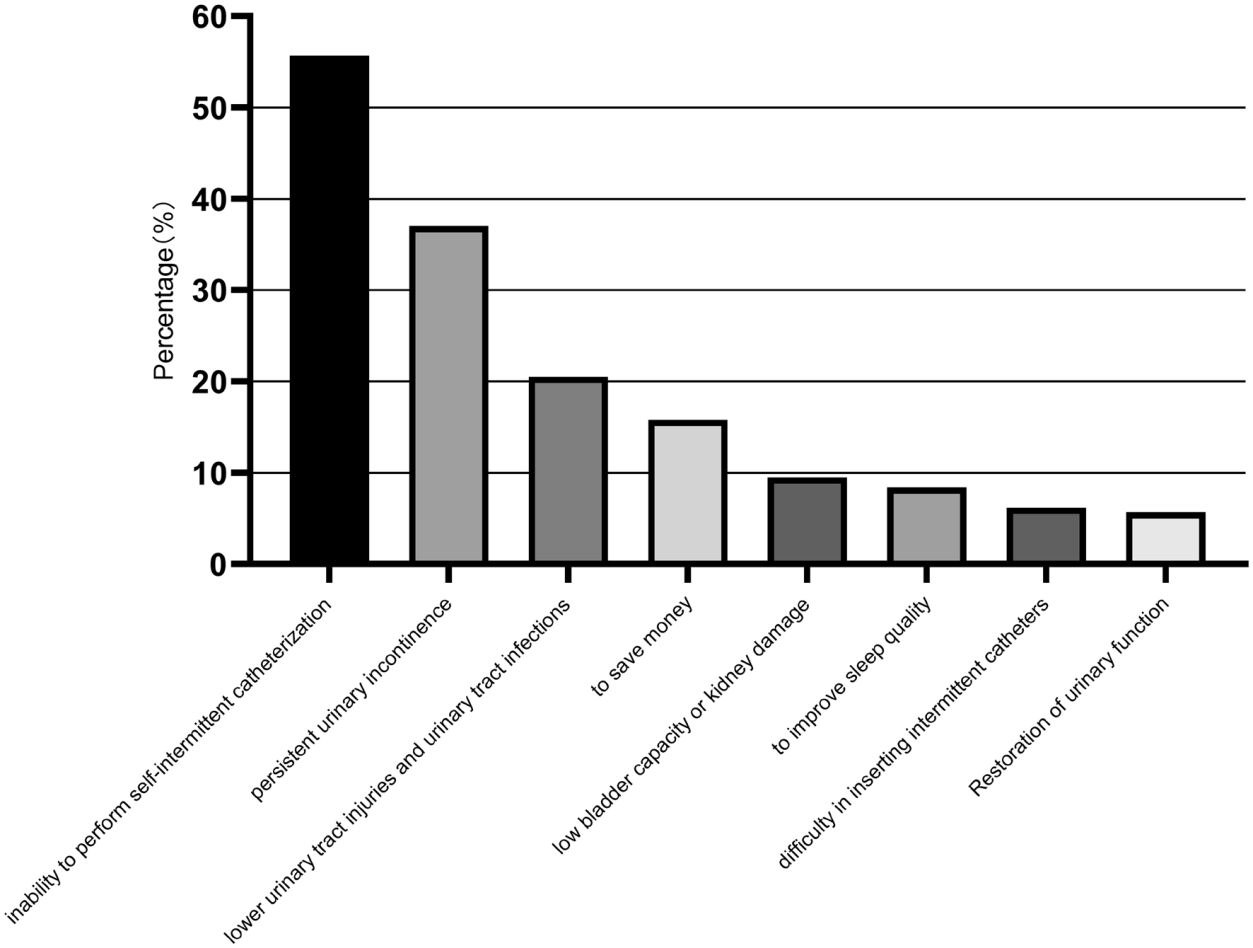
Reasons for discontinuing clean intermittent catheterization (multiple choice).

In the survey, we also asked questions about the details of CIC implementation. Additionally, patients who discontinued CIC during the observation period were asked by telephone about the reasons for discontinuation. Of the 632 CIC patients in the sample, 157 discontinued CIC, and the reasons for and percentage of discontinuation are shown in Fig. 3.

Next, we analyzed patients using CIC separately. As shown in Table 4, the results of univariate analysis indicate that gender, walking ability, frequency of CIC use per day, difficulty of catheter insertion into the urethra, frequency of urinary incontinence per day, changes in catheterization patterns, repeated use of catheters, age and duration of disease were identified as statistically significant factors affecting UTIs in patients with CIC (P < 0.05). The above factors with univariate analysis that yielded statistically significant differences were included in the regression analysis. Table 5 shows the risk factors for UTIs in patients with CIC including male, long duration of disease, difficulty of catheter insertion into the urethra, frequency of urinary incontinence per day ≥ 1, change to other emptying methods and repeated use of catheters.

**Table 4:** Univariate analysis of urinary tract infections in patients with CIC.

Characteristics	Categories	Total (*n* = 623)	Non-UTIs (*n* = 98)	UTIs (*n* = 525)	*P*
		*N* (%)	*N*(%)	*N* (%)	
Age (in years)^a^		44 (18)	40 (17)	45 (18)	0.016
Duration of disease (in months)^a^		81 (156)	59 (89)	86 (172)	0.004
Gender	Male	438 (70.3)	60 (61.2)	378 (72.0)	0.032
	Female	185 (29.7)	38 (38.8)	147 (28.0)	
Educational background	Bachelor's degree and above	58 (9.3)	13 (13.3)	45 (8.6)	0.142
	High School and below	565 (90.7)	85 (86.7)	480 (91.4)	
Etiology	Traumatic	521 (83.6)	87 (88.8)	434 (82.7)	0.134
	Nontraumatic	102 (16.4)	11 (11.2)	91 (17.3)	
Level of nerve damage	Suprasacral	595 (95.5)	95 (96.9)	500 (95.2)	0.456
	Sacral	28 (4.5)	3 (3.1)	25 (4.8)	
Water intake (mL/24 h)	< 1000	205 (32.9)	27 (27.6)	178 (33.9)	0.465
	1000–2000	333 (53.5)	57 (58.2)	276 (52.6)	
	> 2000	85 (13.6)	14 (14.3)	71 (13.5)	
Urine output (mL/24 h)	< 1000	251 (40.3)	40 (40.8)	211 (40.2)	0.053
	1000–2000	274 (44.0)	46 (46.9)	228 (43.4)	
	> 2000	61 (9.8)	3 (3.1)	58 (11.0)	
	Unclear	37 (5.9)	9 (9.2)	28 (5.3)	
Upper limb function	Damaged	187 (30.0)	28 (28.6)	159 (30.3)	0.734
	Normal	436 (70.0)	70 (71.4)	366 (69.7)	
Walking ability	Using wheelchair	588 (94.4)	87 (88.8)	501 (95.4)	0.009
	Independent/assisted walking	35 (3.0)	11 (11.2)	24 (4.6)	
Frequency of CIC use (times per day)	< 1	116 (18.6)	9 (9.2)	107 (20.4)	0.009
	1–6	478 (76.7)	87 (88.8)	391 (74.5)	
	> 6	29(4.7)	2 (2.0)	27 (5.1)	
Difficulty of catheter insertion into the urethra	Without difficulty	420(67.4)	80 (81.6)	340 (64.8)	0.001
	Difficulty	203 (32.6)	18 (18.4)	185 (35.2)	
Frequency of urinary incontinence (times per day)	Never	76 (12.2)	20 (20.4)	56 (10.7)	0.026
	≥ 1	403 (64.7)	57 (58.2)	346 (65.9)	
	< 1	144 (23.1)	21 (21.4)	123 (23.4)	
Changes in catheterization patterns	No change	466 (74.8)	84 (85.7)	382 (72.8)	0.013
	Change to spontaneous voiding	9 (1.4)	2 (2.0)	7 (1.3)	
	Change to other methods	148 (23.8)	12 (12.2)	136 (25.9)	
Repeated use of catheters	Single-use	551 (88.4)	95 (96.9)	456 (86.9)	0.004
	Multiple-use	72 (11.6)	3 (3.1)	69 (13.1)	

UTIs, urinary tract infections; CIC, clean intermittent catheterization;

^a^For For *Age* and *Duration of disease*, report the medians and interquartile range.

**Table 5:** Logistic regression analysis of urinary tract infections in patients with CIC.

Characteristics	Categories	B	SE	OR	*95% CI*	*P*
Gender	Women	Reference				
	Male	0.693	0.248	2.001	1 230–3.254	0.005
Walking ability	Independent/assisted walking	Reference				
	Using wheelchair	0.817	0.416	2.263	1.001–5.120	0.050
Frequency of IC (times per day)	1–6	Reference				
	< 1	0.288	0.412	1.333	0.595–2 988	0.485
	> 6	0.866	0.764	2.378	0.532–10.626	0.257
Age (in years)		0.008	0.009	1.008	0.990–1.026	0.395
Duration of disease (in months)		0.003	0.001	1.003	1.000–1.005	0.025
Difficulty of catheter insertion into the urethra	No	reference				
	Yes	0.832	0.290	2.298	1.302–4.058	0.004
Frequency of urinary incontinence per day	Never	Reference				
	≥ 1	0.773	0.322	2.167	1.152–4.076	0.016
	< 1	0.457	0.386	1.579	0.741–3.365	0.236
Changes in catheterization patterns	No change	Reference				
	Change to spontaneous voiding	− 0.673	0.856	0.510	0.095–2.731	0.432
	Change to other methods	0.858	0.353	2.358	1.180–4.711	0.015
Repeated use of catheters	Single-use	Reference				
	Repeated use	1.166	0.622	3.512	0.948–10.861	0.061

## Discussion

The aim of this study was to understand the current status of UTIs and bladder management in Chinese community-based patients with SCI, and to find ways to improve bladder management to reduce the incidence of UTIs. Based on the extensive questionnaire data, we have reached disturbing results that exceed expectations.

### UTIs current status

A recent literature review reported that UTIs is common in patients with SCI, with a reported global prevalence of 10–68%^21^. The prevalence of UTIs varies in different countries, suggesting that the incidence of UTIs varies widely depending on the healthcare setting and patient characteristics. In our study, the incidence of UTIs in Chinese community SCI patients was 72.9%, which was much higher than the reported data of Chinese in-hospital patients with SCI. In a series of studies of in-hospital patients with SCI, the highest incidence of UTIs was 35%^12^. This suggests that the disease management status of Chinese community SCI patients is different from that of hospitalized patients.

Our results may provide new empirical evidence on the current status of the use of three catheterization methods (CIC, UIC and SPC) in SCI patients in the Chinese community. Among patients with SCI in the Chinese community, the incidence of UTIs was higher in patients using any method of catheterization than in patients who urinated on their own. It is generally accepted that CIC reduces the incidence of UTIs compared with UIC, and both the EAU and AUA recommend CIC as the preferred method of neurogenic bladder emptying^7,22^. In this study, our results are in line with the current EAU and AUA guidelines that the incidence of UTIs in patients with CIC is significantly lower than that in patients with UIC. Conversely, the advantages and disadvantages of CIC compared with SPC have been controversial due to the lack of statistically significant results from relevant studies^23^. Our findings showed that although there was no significant difference in the incidence of UTIs between SPC and CIC patients, the incidence of RUTIs in SPC patients was similar to that of UIC patients and significantly higher than that of UTIs patients. Therefore, among the three methods of urinary catheterization based on UTIs considerations, we preferred to recommend CIC as a bladder emptying method for SCI patients in the Chinese community. However, unfortunately, our findings indicated that compared with SPC and UIC, CIC did not significantly reduce the burden of patients visiting to hospitals or even being hospitalized for UTIs.

### Current status of CIC

Our findings suggest that the proportion of Chinese community spinal cord injury patients using CIC was 23.1%. This result is not only lower than that reported in previous studies on China, but also lower than that reported in other developing countries. Specifically, a 2014 study that followed patients in China within three years of hospital discharge showed that the percentage of these patients using CIC was 44.6%^24^; Bülent et al.^25^ reported that the proportion of Turkish community spinal cord injury patients using CIC was 36.8%; Roop et al.^26^ showed that the proportion of SCI patients using CIC was 34% in India. Both studies have showed a higher proportion of community patients using CIC compared to China. In contrast to these studies, our results using the most recent data from 2020 show that CIC use is even lower among patients in the Chinese community.

Since our data acquisition differed significantly from the aforementioned studies is that we did not use a follow-up approach (observation periods are generally short), but rather opted for a cross-sectional questionnaire approach, which allowed us to obtain data on a sample of patients with a median disease duration of 148 month. Thus, we conjectured that the proportion of Chinese patients using CIC at hospital discharge may decrease as the disease duration increases. Our further analysis of bladder emptying methods in Chinese community SCI patients according to disease duration showed that the proportions of patients with a disease duration of less than 1 year, 1–10 years, and more than 10 years using CIC were 51.4%, 29.6%, and 14.3%, respectively. Since the questionnaire survey in this study was conducted in a highly urbanized area with the best economic and medical conditions in China, the possibility that the reason for obtaining a lower percentage of CIC use was due to the lower prevalence of CIC in China was excluded. In summary, the low percentage of CIC use among community-based SCI patients in China found in this study is most likely due to poor patient adherence to CIC use. This conjecture is supported by the existing studies. For example, Perkash et al.^27^ followed up 50 patients for an average of 22 months and 66% of whom stopped using CIC at follow-up; Afsar et al.^28^ found that patients’ use of CICs at discharge was 63.4%, which declined to 37.5% after an average of 54 months of follow-up.

We have also followed the pioneering studies and have analyzed the reasons for poor CIC adherence. Although Timoney et al.^29,30^ reported that the main reason for low adherence in CIC patients was persistent incontinence, however, in our study, among the reasons (multiple choice) for discontinuing CIC, “inability to perform self-intermittent catheterization” was the highest at 55.7%, followed by “persistent urinary incontinence” at 37%, and “lower urinary tract injuries and urinary tract infections”. Our questionnaire newly added an option that has rarely been taken into consideration in other studies, “to save money”, which came in fourth place at 15.8%.

### Risk factors for UTIs in patients with CIC

Although CIC is the more recommended approach, the proportion of uremia caused by it (84.3%) in the Chinese community cannot be ignored. Despite the fact that the 2003 EAU guidelines recommended CIC as the “gold standard” drainage regimen for the treatment of neurogenic bladder^31^, the promotion and implementation of CIC in China is less than 10 years, and therefore lacks standardized management.

Our analysis revealed the following risk factors for UTIs in SCI patients using CIC in the Chinese community. First, the risk of UTIs in patients using CIC increases with the duration of the disease. We believe that the possible reasons for this are that bladder function in patients with chronic SCI changes over time^32^, as well as the fact that voiding dysfunction after SCI may impede the permeability of the urinary barrier and its sensory function^33^, or the fact that SCI significantly reduces the barrier function of the bladder proteins E-cadherin and Uuroplakin III^34^. Thus, the prolonged course of the disease in patients with SCI makes them more susceptible to UTIs. Second, multiple catheterizations in CIC patients lead to an increased risk of UTIs. While there exists a persistent view, such as that of Kiddoo et al.^35^ that single-use hydrophilic coated catheters may not reduce the incidence of symptomatic UTIs over clean, reused polyvinyl chloride catheters in community-dwelling chronic IC users. However, other studies, such as Vapnek et al.^36^, have also reported that multiple Polyvinylchloride catheters increase the risk of hematuria and significantly increase the incidence of UTIs compared with single-use hydrophilic-coated catheters. Third, we found that switching to other methods of bladder emptying in patients using CIC was also a risk factor for UTIs. This is consistent with existing studies that have concluded that standardized use of CIC reduces the risk of UTIs^37^. Fourth, difficulty in catheter insertion into the urethra contributes to the increased risk of UTIs. Difficulty in catheter insertion can lead to problems such as pain, bleeding, and urethral injury. Correct and appropriate catheter insertion methods are essential for the prevention of UTIs^38^. Fifth, our findings demonstrated that male was also a risk factor for UTI in patients using CIC. However, the effect of gender may be difficult to interpret. Many opinions suggest that it is difficult to compare the effect of gender on complications, including UTIs because males are overrepresented in the SCI population^39,40^. Findings from previously published reports are also quite different. Neither Waites et al.^41^ nor Edokpolo et al.^40^ found sex to be a predictor. However, differences in the incidence of UTI and RUTI between males and females in SCI patients undergoing CIC have been examined and found to be significantly higher in females^42,43^. Conversely, males have been reported to be a significant predictor of febrile UTI in individuals with SCI performing CIC^44^. Last, urinary incontinence is a serious issue faced by most patients with SCI using CIC, and only 12.2% of the patients we surveyed did not have urinary incontinence, which increases the risk of UTIs^45^.

## Limitations

First, the investigation was conducted on patients with SCI in the community. In contrast to studies of in-hospital patients, the determination of UTIs may be controversial due to the lack of laboratory results. Besides, the data for the study were obtained from the results of questionnaires conducted by the patients themselves. Due to the limited expertise of the patients, we had to use plain language as much as possible to ensure that the patients could fully understand the questions and to avoid random responses from patients who did not understand the terminology. Therefore, it is very unfortunate that there was no way to obtain urodynamic data as well as standard neurological classification of SCI by the American Spinal Injury Association Impairment Scale. Moreover, it is challenging to achieve precise classification when distinguishing between various voiding techniques, particularly when confronted with situations characterized by continuous and dynamic alterations.

## Conclusion

Overall, our findings suggest that Chinese community SCI patients have a higher prevalence of UTIs compared to Chinese in-hospital and out-of-hospital patients in medically developed countries. In addition, we found that CIC had the lowest incidence of UTIs among the three methods of catheterization, and that SCI patients with CIC had low rates of use and poor compliance. Further analyses showed that most of the risk factors for UTIs in CIC patients were associated with irregular CIC use. Therefore, in response to the above findings, we obtained the following insights. First, when choosing a bladder emptying method for SCI patients in the Chinese community, CIC is the preferred method for patients who are unable to void on their own. Second, stronger caregiver support and financial support are needed to improve CIC compliance. Finally, we call for a database of Chinese SCI patients in order to receive information about patients' diseases more quickly and to enable closer contact between doctors and patients. We believe that by strengthening the management of bladder emptying methods and further standardizing CIC operations, the risk of urosepsis in SCI patients in the Chinese community will be greatly reduced in the foreseeable future.

## Data Availability

The data that support the findings of this study are available from the corresponding author upon reasonable request.
